# Comparison of Postoperative Pain and Adverse Effects between Variable-Rate Feedback Infusion and Conventional Fixed-Rate Basal Infusion Modes of Patient-Controlled Epidural Analgesia Following Open Gastrectomy: A Randomized Controlled Trial

**DOI:** 10.3390/ijerph18168777

**Published:** 2021-08-19

**Authors:** Yoo Kyung Jang, Na Young Kim, Jeong Soo Lee, Hye Jung Shin, Hyoung Gyun Kim, Suk Woo Lee, Jae Chul Koh, Young Chul Yoo

**Affiliations:** 1 Department of Anesthesiology and Pain Medicine, Korea University College of Medicine, 73 Goryeodae-ro, Seongbuk-gu, Seoul 02841, Korea; gamtang@korea.ac.kr (Y.K.J.); yjgim0912@naver.com (H.G.K.); sunday249@gmail.com (S.W.L.); 2 Department of Anesthesiology and Pain Medicine, Anesthesia and Pain Research Institute, Yonsei University College of Medicine, 50-1 Yonsei-ro, Seodaemun-gu, Seoul 03722, Korea; knnyyy@yuhs.ac (N.Y.K.); ration99@yuhs.ac (J.S.L.); 3 Biostatistics Collaboration Unit, Yonsei University College of Medicine, 50-1 Yonsei-ro, Seodaemun-gu, Seoul 03722, Korea; hjshin105@yuhs.ac

**Keywords:** postoperative pain, postoperative nausea, background infusion, patient-controlled epidural analgesia, open gastrectomy

## Abstract

Patient-controlled epidural analgesia is widely used to control postoperative pain following major intra-abdominal surgeries. However, determining the optimal infusion dose that can produce effective analgesia while reducing side effects remains a task to be solved. Postoperative pain and adverse effects between variable-rate feedback infusion (VFIM group, *n* = 36) and conventional fixed-rate basal infusion (CFIM group, *n* = 36) of fentanyl/ropivacaine-based patient-controlled epidural analgesia were evaluated. In the CFIM group, the basal infusion rate was fixed (5 mL/h), whereas, in the VFIM group, the basal infusion rate was increased by 0.5 mL/h each time a bolus dose was administered and decreased by 0.3 mL/h when a bolus dose was not administered for 2 h. Patients in the VFIM group experienced significantly less pain at one to six hours after surgery than those in the CFIM group. Further, the number of patients who suffered from postoperative nausea was significantly lower in the VFIM group than in the CFIM group until six hours after surgery. The variable-rate feedback infusion mode of patient-controlled epidural analgesia may provide better analgesia accompanied with significantly less nausea in the early postoperative period than the conventional fixed-rate basal infusion mode following open gastrectomy.

## 1. Introduction

Patient-controlled epidural analgesia (PCEA) has been widely used to control postoperative pain following major intra-abdominal surgeries [1,2]. It is well known that patients who undergo open gastrectomy often experience substantial postoperative pain; thus, management of adequate pain is essential to facilitate early postoperative recovery [3]. It is also known that administration of epidural local anesthetics with opioids to patients undergoing abdominal surgery reduces postoperative pain when compared with that of systemic or epidural opioids or epidural local anesthetics alone [4].

For PCEA, a demand dose with or without continuous basal infusion is commonly used [5]. However, a demand dose mode without basal infusion is difficult to control the breakthrough pain that occurs when the patient coughs or moves [6]. In addition, it may not be effective while sleeping because it relies on the patient’s intention to press the button [7]. Moreover, the number of analgesics consumed may increase [8]. Therefore, PCEA using a demand dose with a continuous basal infusion at a constant rate has been widely used. However, determining the appropriate basal infusion rate for PCEA remains controversial. If a basal infusion is set lower than the requirement, analgesia may be insufficient. In contrast, side effects such as postoperative nausea and vomiting (PONV), sedation, dizziness, hypotension, and respiratory depression may occur when the infusion rate is too high [9,10].

Determining an optimal infusion dose that can produce effective analgesia while reducing side effects remains a task to be solved. Since the patient-related factors that determine the pharmacokinetic/dynamics of PCEA drugs, such as body mass index (BMI), sex, fat proportion, and age, are very diverse, it is challenging to determine the optimal infusion dose [11,12,13].

Recently, a new PCA device that comprises an “optimizing background infusion mode” was introduced, which can adjust the background infusion rate depending on bolus demand over a predefined time [14,15]. However, this mode is yet to be applied in PCEA for postoperative pain control, and there is a dearth of literature on its efficacy and adverse effects [8,14,15].

The aim of the present study is to compare the postoperative analgesic efficacy and incidence of nausea between the variable-rate feedback infusion mode (VFIM) and conventional fixed-rate infusion mode (CFIM) of ropivacaine/fentanyl-based PCEA in patients who underwent open gastrectomy.

## 2. Materials and Methods

### 2.1. Study Population

This prospective, randomized controlled trial was approved by the Institutional Review Board (IRB) and Hospital Research Ethics Committee of Severance Hospital, Yonsei University Health System, Seoul, Korea (IRB protocol No. 4-2016-1152). The trial was registered at the ClinicalTrials.gov Protocol Registration System (registration no. NCT03430440). Written informed consent was obtained from all participants, and the study was conducted in accordance with the principles of the Helsinki Declaration.

A total of 76 American Society of Anesthesiologists (ASA) physical status I-III patients (aged 20–70 years), who were scheduled for elective open gastrectomy, were enrolled between December 2017 and January 2019. The exclusion criteria were history of hematologic clotting defects, sepsis, distant metastasis, allergy to PCEA drugs (opioids and local anesthetics), and pregnancy or lactation.

### 2.2. Anesthetic Management

In the pre-anesthetic room, all enrolled patients were explained how to convey pain intensity using a numerical rating scale (NRS; 0, no pain, and 10, worst pain possible), and on how to use the PCEA device (PAINSTOP[PS-1000], Unimedics Co., Seoul, Korea) [16].

After transfer to the operating room, the blood pressure and pulse oximetry, and electrocardiogram parameters were monitored in patients of both groups using a standard monitor (Patient monitor M1205A, Philips, USA or Micro O2, Siemens, Germany). Insertion of the epidural catheter was performed prior to the induction of general anesthesia. At the T8/9 or T9/10 level, the catheter was advanced 5 cm cephalad through the epidural space via a 17-gauge Touhy needle (Portex^®^ Combined Spinal/Epidural Minipack with Lock, pencil-point spinal needle, Smiths Med Int Ltd., Hythe, UK). After confirming that blood or spinal fluid did not flow back through the epidural catheter, 3 mL of 1% lidocaine (Daihan Lidocaine HCl 2% inj, Daihan, Seoul, Korea) was administered with the epidural catheter, and sensory blockade was confirmed using a pinprick test.

Following intravenous administration of 0.2 mg of glycopyrrolate (Glycopyrrolate injection, Reyon Pharm. Co. Ltd. Seoul, Korea), 1.5–2 mg/kg of propofol (Fresofol^®^ MCT 1%, Fresenius Kabi Korea Ltd., Seoul, Korea), 0.5–1 µg/kg of remifentanil (Ultian injection, Hanlim Pharm. Co. Ltd., Seoul, Korea), and 0.6 mg/kg of rocuronium (Rocnium injection, Hanlim Pharm. Co. Ltd., Seoul, Korea) were used to induce anesthesia.

Endotracheal intubation was performed when the patient’s reflex was absent. For mechanical ventilation using a ventilator (Primus, Dräger Medical, Lübeck, Germany), the following settings were used: tidal volume of 8 mL/kg, a positive end-expiratory pressure of 5 cmH_2_O, and a respiratory rate to maintain the end-tidal carbon dioxide level between 35 and 40 mmHg and oxygen saturation higher than 99% in 50% O_2_/air. The maintenance of anesthesia was adjusted with 0.7–1.2 age-adjusted minimal alveolar concentration of desflurane (Suprane^®^, Baxter Healthcare, Deerfield, IL, USA) and remifentanil 0.05–0.1 µg/kg/min by targeting bispectral index (BIS) monitoring (Aspect A-2000; Aspect Medical System Inc., Newton, MA, USA) scores at 40–60. The anesthetic agents were titrated to maintain the mean blood pressure (MBP) and heart rate (HR) within 25% of baseline values and to provide an appropriate depth of anesthesia. Intraoperative fluid was maintained using crystalloid fluid (Plasma solution A, CJ Pharmaceutical, Seoul, Korea) at a constant rate of 5–10 mL/kg/h. Hypotension (MBP < 60 mm Hg) was managed with intravenous ephedrine (Ephedrine HCl^®^, Daewon Pharmaceuticals, Seoul, Korea) at 4 mg increments, and 0.25 mg intravenous atropine (Daehan Atropine, Daehan Pharmaceutical, Seoul, Korea) was used to manage bradycardia (HR < 40 beats/min). At the end of the surgery, 0.2 mg of glycopyrrolate and 1.0 mg of neostigmine (neostigmine methylsulfate injection, Daihan Pharm. Co. Ltd. Seoul, Korea) were administered to reverse neuromuscular blockade.

### 2.3. Randomization and Intervention

After enrollment, the patients were randomly assigned to two groups that used a PAINSTOP device applying either the conventional fixed-rate basal infusion mode (CFIM group, *n* = 38) or the variable-rate feedback infusion mode (VFIM group, *n* = 38) using a computer-generated random table with no dividing blocks and stratification.

The PCEA regimen was a mixture of a total volume of 250 mL, comprising 0.15% ropivacaine (Nacain Injection, Huons Co., Sungnam, Korea), 15 µg/kg of fentanyl (fentanyl citrate injection, Hana Pharm Co. Ltd., Seoul, Korea), and normal saline in both groups. All PCA devices were set to administer a bolus of 0.5 mL (fentanyl: 0.03 µg/kg) with a lockout interval of 15 min and a basal infusion rate of 5 mL/h (fentanyl: 0.3 µg/kg/h). The basal infusion rate in the VFIM group was set to increase automatically by 0.5 mL/h (fentanyl: 0.03 µg/kg/h) each time a bolus dose was administered by pushing the button and decrease by 0.3 mL/h (fentanyl: 0.018 µg/kg/h) when a bolus dose was not administered for two hours. The basal infusion rate was specified as a maximum flow rate of 7.5 mL/h (fentanyl: 0.45 µg/kg/h) and a minimum flow rate of 3 mL/h (fentanyl: 0.18 µg/kg/h). At the initiation of peritoneal closure, the PCA device was commenced according to the group assignment following the bolus injection of 5 mL of ropivacaine 0.15% via the epidural catheter and 0.3 mg of intravenous ramosetron (Nasea injection, Astellas Pharma Korea Inc., Seoul, Korea) for PONV.

Following the patients were arrived at the recovery room after surgery, instructions on the use of the PCEA device were repeated. Patients were encouraged to press the bolus button whenever the resting NRS score was 3 or higher. Recovery nurses who were not part of the study assessed the resting NRS score, and additional rescue analgesics with fentanyl at 0.5 µg/kg increments were administered to patients who experienced sustained pain at a resining NRS score of >4 in the recovery room.

After discharge from the recovery room, postoperative pain, any adverse effects related to PCEA, and the amounts of the consumed volume of PCEA were assessed at 1, 6, 24, 48 h after surgery. Similarly, for patients who experienced sustained pain at a resting NRS score of >4 in the admission room, pethidine (Pethidine HCL, Jeil Pharm, Daegu, Korea) was administered at increments of 12.5 mg.

### 2.4. Data Collection

All data were collected prospectively. These included the registered demographics and intraoperative variables such as age, BMI, sex, ASA physical status, underlying co-morbidities, type of gastrectomy, duration of anesthesia and operation, administered amount of fluid intake, amount of blood loss, urine output, and dose of administered intraoperative remifentanil and ephedrine. Intraoperative hemodynamic variables, including MBP, HR, and BIS were also collected during the intraoperative period. The resting NRS score; the amount of consumed volume of PCEA; the number of patients who received additional rescue analgesics; and those who experienced any PCEA-related adverse effects including nausea, vomiting, dizziness, hypotension, tachypnea, numbness, and pruritus were assessed at 1, 6, 24, and 48 h after surgery. In addition, the duration of postoperative hospital stays and the discontinuation rate of PCEA were evaluated.

### 2.5. Statistical Analysis

The primary endpoint of the current study was the resting pain intensity at six hours after surgery. Based on a preliminary study, the resting NRS score at six hours after surgery in the CFIM group was 4.05 ± 0.8 and 3.4 ± 0.5 in the VFIM group. Thirty-two patients in each group were required in order to guarantee the power of 90% at a significance level of 5% for a difference of 0.65 in the resting NRS (standard deviation of 0.8). Considering a 15% dropout rate, 38 patients were recruited in each group.

For continuous variables, the Shapiro–Wilk test was used to test the normality of data distribution. Statistical analysis was performed using Student’s t-test for normally distributed continuous variables, including weight, BMI, and the duration of anesthesia and surgery; these variables were presented as mean ± SD. For continuous variables that were not normally distributed, including age; fluid intake; amount of blood loss; urine output; intraoperative dose of remifentanil and ephedrine; intraoperative MBP, HR, and BIS; postoperative NRS score; volume of PCEA consumed; and duration of postoperative hospital stay, the Mann-Whitney U test was used to determine whether there was a significant difference between the two groups, and data were presented as median [Q1, Q3]. For categorical variables, including sex, ASA physical status, underlying diseases such as hypertension and diabetes, type of gastrectomy, number of patients who received additional rescue analgesics, number of patients who experienced any PCEA-related adverse effects, and the discontinuation rate of PCEA, the data were presented as frequencies (%), and significant relationships between groups and variables were determined using the chi-square test. In the contingency table of groups and variables, if an expected frequency of less than 5 occurred in more than 20% of the cells, Fisher’s exact test was used. Statistical significance was set at *p* < 0.05. All statistical analyses were conducted by SAS (version 9.4; Cary, NC, USA) and R software (version 4.04; R Foundation for Statistical Computing, Vienna, Austria).

## 3. Results

**Figure 1:**
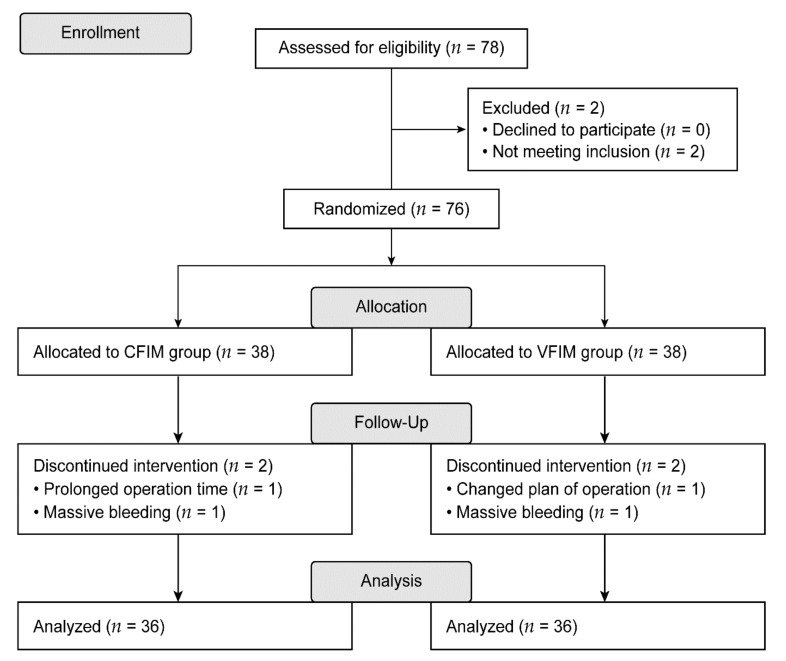
Flow diagram depicting patient enrollment. CFIM, conventional fixed-rate infusion mode; VFIM, variable-rate feedback infusion mode.

Among the 78 patients, two who did not meet the inclusion criteria were excluded. Thus, 76 patients were finally enrolled, and these were randomly allocated to each of the two groups. Following enrollment, discontinued intervention occurred for two patients in each group: two in the CFIM group owing to a prolonged operation time and massive bleeding, and two in the VFIM group owing to a change in the operation plan and massive bleeding. Thus, a total of 36 patients in each group were included for the final analysis (Figure 1).

The demographic and intraoperative characteristics of the enrolled patients are presented in Table 1. There were no significant differences in demographic data, medical history, duration, and doses of drugs used during anesthesia between the two groups. No significant differences were observed in intraoperative MBP, HR, and BIS between the two groups.

The NRS, total administered dose of PCEA, and the number of patients who received rescue opioids at each time point are shown in Table 2. No differences were observed in the total administered dose of PCEA and the number of patients who received the additional opioids at each time point between the two groups.

**Figure 2:**
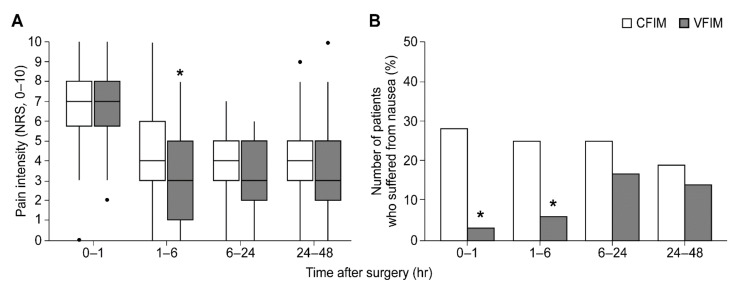
Resting numeric rating score of pain intensity (**A**) and the number of patients who experienced postoperative nausea (**B**). Box plot with median (solid line), interquartile range (box), and values within 1.5 times the interquartile range (whiskers) are shown. * *p* < 0.05. NRS, numeric rating score; CFIM, conventional fixed-rate infusion mode; VFIM, variable-rate feedback infusion mode.

At one to six hours after surgery, patients in the VFIM group had significantly less pain than those in the CFIM group (4 [3, 6] vs. 3 [1, 5], respectively; *p* = 0.031) (Table 2 and Figure 2A). However, there were no significant differences in the resting pain intensity between the two groups at other time intervals. The number of patients who suffered from postoperative nausea was significantly lower in the VFIM group than in the CFIM group until six hours after surgery (Figure 2B and Table 3). However, at other time intervals, no significant differences were observed between the two groups.

The data on the duration of postoperative hospital stays and PCEA-related adverse effects following surgery are summarized in Table 3.

As seen from the table, the postoperative hospital stay was not significantly different between the groups. Other PCEA-related adverse effects such as vomiting, dizziness, hypotension, tachypnea, numbness, and pruritus were also not significantly different between the two groups. In addition, with respect to the rate of discontinuation of PCEA, no difference was observed between the groups (one each with hypotension and uncontrolled nausea in the CFIM group vs. two with hypotension and one with uncontrolled fever in the VFIM group; *p* > 0.999).

## 4. Discussion

This prospective randomized controlled trial demonstrated that a VFIM of PCEA may provide better analgesia that is accompanied by significantly less nausea in six hours after surgery than CFIM in patients after open gastrectomy.

Determining an appropriate infusion rate requires consideration of several factors including BMI, sex, and age, which can influence the efficacy of PCEA [11,13,17,18]. In addition, the degree of pain and demand for analgesics may change over time after surgery. Generally, postoperative pain intensity is more severe on the day of surgery; the degree and frequency decrease over time [19,20]. Therefore, background infusion of a constant fixed-rate dose may not effectively meet these needs. However, a variable-rate feedback infusion dose change mode may compensate for these disadvantages by reflecting patient demand.

In the present study, the patients in the VFIM group reported significantly less resting pain intensity at one to six hours after surgery, which was the primary endpoint of the study. However, there was no significant difference in the resting NRS score between the two groups immediately after arriving at the recovery room. The dose infused in the recovery room may not have had enough time to make a difference in the analgesic effect depending on the PCEA background dose change modality. The bolus injection of a local anesthetic at the end of the surgery would have provided similar analgesic effects in both groups, and residual anesthesia may have been affected. It is likely that the significant difference in the resting pain intensity at one to six hours after surgery was affected by the difference in the background infusion dose change modality.

There are few reports that have documented the usefulness of variable-rate feedback infusion control of the basal infusion rate in epidural analgesia. Generally, it is considered that higher analgesic doses of PCEA may provide better analgesia; however, previous studies have reported that higher analgesic doses of PCEA are not always associated with better pain control [21]. Pain is defined as “an unpleasant sensory and emotional experience associated with or resembling that associated with actual or potential tissue damage” [22]. Such pain has many subjective elements, and these subjective emotions may respond sensitively to changes in the intensity of stimuli [23]. Therefore, appropriate analgesic dose changes in response to changing pain stimuli may lead to better pain control. In the present study, the dose of analgesic increased more steeply in the early postoperative period and then gradually decreased in the VFIM group. It is possible that patients in this group would have responded more effectively to the stimulation of postoperative pain, which is generally more severe in the early postoperative period.

Pain threshold can be affected by several factors, such as age, sex, and psychological factors [17,24,25]. It is very difficult to determine the most effective basal infusion dose by considering only these factors. In contrast, adjusting the dose by reflecting the patient’s requirements can lead to a reduced probability of using the inadequate analgesic dose, which has been demonstrated in the present study as a better early postoperative analgesia.

PCEA is associated with the development of several adverse effects [1,11]. Among these, nausea is one of the most common complications [1]. The chemoreceptor-triggering zone, which is located within the area postrema, is known to induce nausea and vomiting reactions by detecting substances potentially harmful to the body [26]. Administration of opioids or local anesthetics into the epidural space can often cause nausea by stimulating this mechanism. Many risk factors for PONV have been identified [26,27]. As mentioned above, it might be difficult to determine the appropriate basal infusion dose by considering all these factors. However, adjustment of the infusion rate according to the pain intensity of the patient may have contributed to the amount of titration of the infusion after reflecting the different characteristics of each patient. Although further research is required, a possible explanation for the significantly lower incidence of nausea in the VFIM group in the present study may be attributed to the reduction in the number of patients receiving excessive chemical stimuli.

It has been documented that the intensity of postoperative pain also affects the incidence of nausea [28,29]. It has been shown that pain is a major risk factor for nausea than the administration of analgesics [29]. Pain itself induces general arousal of the central nervous system, which can lead to vomiting by other stimuli. In addition, activation of nociceptors owing to pain can cause changes in the central nervous system, the vomiting threshold, and the chemoreceptor trigger zone [29]. In the present study, compared with the CFIM group, the VFIM group had significantly less nausea in accordance with the significantly lower resting NRS during the early postoperative period (one to six hours). This may be another explanation for this mechanism.

The study has some limitations. First, only Korean patients with gastric cancer who underwent open gastrectomy were enrolled. Consequently, the study was conducted on a sample with an unintentionally narrow age and gender distribution. Therefore, the results of this study are difficult to be applied to patients with different characteristics or undergoing different surgeries. In addition, the small sample size might not have been sufficient to discriminate the difference in pain intensity or adverse effects at other time intervals. With a larger sample size, perhaps, it might have been possible to compare more accurately as to whether there were any differences in other adverse effects or in other time intervals. Nevertheless, this study is meaningful in showing the possibility that basal infusion control according to the patient’s needs with the epidural route can be an effective approach.

## 5. Conclusions

Our study shows that VFIM, in which the basal rate was adjusted according to the administration of a bolus dose, provided better analgesia with less nausea in the early postoperative period than the CFIM of fentanyl/ropivacaine-based PCEA after open gastrectomy.

## Figures and Tables

**Table 1:** Demographic and intraoperative variables.

	CFIM Group (*n* = 36)	VFIM Group (*n* = 36)	*p* Value
Age, years	57.5 [51, 60]	56.5 [49, 61]	0.969
Body mass index, kg/m^2^	22.5 ± 3.2	23.6 ± 2.8	0.114
Male sex	22 (61%)	27 (75%)	0.206
ASA physical status, I/II/III	21/14/1	18/18/0	0.343
Comorbidities			
Hypertension	5 (14%)	8 (22%)	0.358
Diabetes mellitus	6 (17%)	4 (11%)	0.496
Subtotal/Total	25/11	26/10	0.795
Anesthesia time, min	182.7 ± 32.8	174.4 ± 31.6	0.278
Operation time, min	145.7 ± 32.8	138.2 ± 32.6	0.403
Fluid intake, mL	1200 [1050, 1400]	1200 [1000, 1500]	0.796
Blood loss, mL	135 [70, 235]	100 [77.5, 200]	0.578
Urine output, mL	157.5 [110, 280]	142.5 [97.5, 250]	0.325
Administered amounts of remifentanil, µg	524 [432, 681]	513 [433, 576]	0.450
Administered amounts of ephedrine, mg	6 [0, 8]	4 [0, 12]	0.862
Mean blood pressure, mmHg			
0 min	89.1 ± 15.5	85.8 ± 13.7	0.346
30 min	87.3 ± 12.7	86.2 ± 11.8	0.707
60 min	92.1 ± 11.6	87.3 ± 9.9	0.060
90 min	88.3 ± 12.4	85.2 ± 9.0	0.253
Heart rate, bpm			
0 min	72 [63, 79]	66 [63, 74]	0.245
30 min	80 [70, 89]	79 [66, 86]	0.456
60 min	75 [67, 82]	71 [65, 78]	0.195
90 min	73 [67, 77]	68 [63, 74]	0.056
Bispectral index			
0 min	43 [36, 56]	40 [35, 46]	0.247
30 min	40 [33, 46]	38 [35, 42]	0.298
60 min	38 [30, 46]	35 [30, 40]	0.111
90 min	35 [28, 43]	32 [29, 36]	0.272

Data are presented as mean ± standard deviation, median [Interquartile range], or number of patients (proportion). ASA, American Society of Anesthesiologists; Subtotal, subtotal gastrectomy; Total, total gastrectomy; CFIM, conventional fixed-rate infusion mode; VFIM, variable-rate feedback infusion mode.

**Table 2:** Postoperative pain-related profile up to 48 h after surgery.

	CFIM Group (*n* = 36)	VFIM Group (*n* = 36)	*p* Value
NRS			
0–1 h	7 [5.5, 8]	7 [5.5, 8]	0.613
1–6 h	4 [3, 6]	3 [1, 5]	0.031 *
6–24 h	4 [3, 5]	3 [2, 5]	0.137
24–48 h	4 [3, 5]	3 [2, 5]	0.538
Administered dose of PCEA			
0–1 h	6.08 [5.37, 7.69]	6.24 [5.49, 7.35]	0.503
1–6 h	27.4 [19.31, 32.54]	28.32 [17.19, 36]	0.305
6–24 h	97.74 [86.4, 108.29]	99.16 [84.04, 122.06]	0.540
24–48 h	215.92 [204.47, 227.84]	207.15 [166.73, 250]	0.953
Number of patients who received rescue opioids			
0–1 h	28 (78%)	22 (61%)	0.125
1–6 h	22 (61%)	19 (53%)	0.475
6–24 h	20 (56%)	17 (47%)	0.479
24–48 h	14 (39%)	17 (47%)	0.475

Data are presented as median [Interquartile range] or number of patients (proportion). * *p* < 0.05. NRS, numeric rating score; PCEA, patient controlled epidural analgesia; CFIM, conventional fixed-rate infusion mode; VFIM, variable-rate feedback infusion mode.

**Table 3:** Postoperative adverse effects up to 48 h after surgery.

	CFIM Group (*n* = 36)	VFIM Group (*n* = 36)	*p* Value
Postoperative hospital stays, days	7 [6, 7.5]	7 [6, 8]	0.394
Nausea			
0–1 h	10 (28%)	1 (3%)	0.003 *
1–6 h	9 (25%)	2 (6%)	0.022 *
6–24 h	9 (25%)	6 (17%)	0.384
24–48 h	7 (19%)	5 (14%)	0.527
Vomiting			
0–1 h	1 (3%)	0 (0%)	>0.999
1–6 h	0 (0%)	0 (0%)	-
6–24 h	0 (0%)	1 (3%)	>0.999
24–48 h	0 (0%)	0 (0%)	-
Dizziness			
0–1 h	2 (6%)	1 (3%)	>0.999
1–6 h	2 (6%)	1 (3%)	>0.999
6–24 h	2 (6%)	5 (14%)	0.429
24–48 h	4 (11%)	4 (11%)	>0.999
Hypotension			
0–1 h	2 (6%)	0 (0%)	0.493
1–6 h	1 (3%)	1 (3%)	>0.999
6–24 h	2 (6%)	7 (19%)	0.151
24–48 h	1 (3%)	3 (8%)	0.614
Tachypnea			
0–1 h	0 (0%)	2 (6%)	0.493
1–6 h	0 (0%)	1 (3%)	>0.999
6–24 h	0 (0%)	0 (0%)	-
24–48 h	0 (0%)	0 (0%)	-
Numbness			
0–1 h	0 (0%)	0 (0%)	-
1–6 h	1 (3%)	0 (0%)	>0.999
6–24 h	1 (3%)	0 (0%)	>0.999
24–48 h	0 (0%)	0 (0%)	-
Pruritus			
0–1 h	0 (0%)	0 (0%)	-
1–6 h	0 (0%)	0 (0%)	-
6–24 h	0 (0%)	0 (0%)	-
24–48 h	1 (3%)	1 (3%)	>0.999
Discontinuation of PCEA	2 (6%)	3 (8%)	>0.999

Data are presented as median [Interquartile range] or number of patients (proportion). * *p* < 0.05. CFIM, conventional fixed-rate infusion mode; VFIM, variable-rate feedback infusion mode.

## Data Availability

The datasets used and/or analyzed during the current study are available from the corresponding author on reasonable request.
